# Editorial: Novel bioconversion technology of waste and wastewater for resource recovery

**DOI:** 10.3389/fmicb.2026.1990924

**Published:** 2026-09-17

**Authors:** Jinsong Liang, Lan Wu, Le Chen, Li Sun

**Affiliations:** 1 Northeast Institute of Geography and Agroecology, Chinese Academy of Sciences (CAS), Harbin, China; 2 School of Civil and Environmental Engineering, The University of New South Wales, Sydney, NSW, Australia; 3 Guangzhou Institute of Energy Conversion, Chinese Academy of Sciences Guangzhou, Guangzhou, China; 4 School of Energy and Environmental Engineering, Hebei University of Technology, Tianjin, China

**Keywords:** bioconversion, biomass waste, photosynthetic bacteria, resource recovery, wastewater

## Introduction

The rapid expansion of industrial and agricultural production worldwide has led to massive accumulations of solid waste and organic wastewater, triggering severe resource wastage and ecological pressure (Zhang et al., 2025). Global solid waste production continues to rise steadily and is projected to increase substantially by 2050, while larges of organic-rich wastewater are continuously discharged from agricultural and industrial activities (Cook et al., 2026). Traditional waste and wastewater disposal strategies primarily focus on pollutant elimination and harmless treatment, yet they neglect the abundant valuable resources embedded in waste streams (Gajera et al., 2026). Such conventional management patterns not only generate considerable greenhouse gas emissions and secondary environmental pollution but also break the circular economy chain of waste resource recovery (Chavan et al., 2022; Mustapha et al., 2026).

Microbial bioconversion provides a promising pathway for addressing these challenges by harnessing the metabolic capacity and functional diversity of microorganisms to transform waste and wastewater into value-added products. However, practical implementation remains constrained by inefficient resource conversion, unstable microbial activity, and limited adaptability to complex environmental conditions. This Research Topic focuses on novel bioconversion technologies for waste and wastewater resource recovery, brings together recent advances in microbial bioconversion and bioresource recovery, with particular emphasis on functional microorganisms, microbial communities, metabolic regulation, and biofilm-based processes.

## Bioplastic production from biomass waste

Zhao W. et al. explored the enhancement effects of Fe^3+^ and Mn^2+^ on polyhydroxybutyrate (PHB) production by photosynthetic bacteria (PSB) using kitchen waste digestate. The optimal ion concentrations effectively improved PSB biomass and PHB accumulation while achieving high pollutant removal efficiency. Furthermore, metal ions reshaped microbial community structure and enhanced core metabolic functions, providing a novel strategy for efficient PHB production from food waste.

Tao et al. reviewed polyhydroxyalkanoate (PHA) production using food waste-derived volatile fatty acids as low-cost carbon sources. The review summarized microbial metabolic mechanisms, key processing strategies, and major influencing factors, while evaluating economic and environmental feasibility and highlighting pathways toward process optimization and large-scale application.

## Efficient isolation of functional microorganisms

Li, Wang et al. employed immobilized *Alcaligenes faecalis* to enhance nitrogen removal in ecological floating islands treating eutrophic wastewater. Optimal C/N and dissolved oxygen conditions were identified, and microbial community analysis revealed key functional taxa under effective operating conditions. Suitable plant combinations were also screened, providing a practical strategy for improving ecological floating island performance.

Li, Zou et al. screened and identified a efficient esterifying strain of *Acinetobacter baumannii* from *Daqu* for the resource utilization of brewing Huangshui. This strain presented excellent ethanol- and acid-tolerance and favorable esterification performance. Esterification treatment of Huangshui using this strain significantly increased ethyl caproate content and reduced ethyl lactate level.

Jiang et al. isolated phosphate-solubilizing bacteria from farmland rhizosphere soil with strong heavy-metal tolerance. Their phosphorus-solubilizing capacity and resistance to Pb and Cr were systematically evaluated, and optimal cultivation conditions were identified, providing promising microbial resources for phosphorus activation and bioremediation of contaminated soils.

## Microbial diversity and community composition

Seth et al. investigated microbial functional dynamics in a long-term, energy-free constructed wetland for urban sewage remediation. Based on 16S rRNA sequencing, distinct microbial communities and functional niches were identified across system components, revealing microbial interactions that support stable wetland performance and providing insights for optimizing nature-based wastewater treatment.

Tonanzi et al. investigated short-chain fatty acid production through anaerobic fermentation of tannery sludge under different temperatures, hydraulic retention times, and pretreatment conditions. Distinct microbial communities dominated under mesophilic oxidative and thermophilic thermal conditions, while longer hydraulic retention times enhanced organic conversion and microbial activity, supporting sustainable tannery sludge valorization.

## Driving mechanisms of biofilms for wastewater treatment

Chen et al. developed an algae-bacteria membrane-aerated biofilm reactor (AB-MABR) to improve nitrogen removal from low-C/N wastewater. Compared with a conventional bacterial MABR, AB-MABR achieved enhanced COD, ammonium, and total nitrogen removal by increasing microbial metabolic activity, extracellular polymeric substance production, and biofilm structural stability. Metagenomic analysis further revealed enhanced metabolic and environmental adaptation pathways, demonstrating the synergistic role of algae and bacteria in carbon-limited wastewater treatment.

Zhao D. et al. developed a combined coagulation-Fenton and multi-stage anaerobic-aerobic biofilm system for treating refractory petrochemical wastewater. Optimized Fenton pretreatment effectively reduced influent COD, while the staged biofilm process maintained stable pollutant removal. This integrated strategy achieved excellent COD elimination, providing a robust technical route for high-strength industrial wastewater treatment.

## Future directions

This Research Topic highlights the great potential of microbial conversion and bioresource recovery for sustainable waste and wastewater remediation. Future studies should focus on scaling lab-based technologies to practical field applications. Further efforts are also required to optimize microbial synergistic mechanisms and develop tailored strategies to improve resource utilization efficiency and environmental adaptability under complex actual conditions.
